# Membranous urethral length is the single independent predictor of urinary continence recovery at 12 months following Retzius-sparing robot-assisted radical prostatectomy

**DOI:** 10.1007/s11701-024-01986-8

**Published:** 2024-05-29

**Authors:** Jorge Fonseca, Maria Francisca Moraes-Fontes, Inês Sousa, Francisco Oliveira, Gonçalo Froes, Ana Gaivão, Artur Palmas, Jorge Rebola, Ciprian Muresan, Tiago Santos, Daniela Dias, Mário Varandas, Antonio Lopez-Beltran, Ricardo Ribeiro, Avelino Fraga

**Affiliations:** 1 Unidade de Próstata, Centro Clínico Champalimaud, Champalimaud Foundation, Av. Brasília, 1400-038 Lisboa, Portugal; 2 Unidade de Imuno-Oncologia, Centro Clínico Champalimaud, Champalimaud Foundation, Lisbon, Portugal; 3 Unidade de Investigação Clínica, Centro Clínico Champalimaud, Champalimaud Foundation, Lisbon, Portugal; 4 Serviço de Medicina Nuclear, Centro Clínico Champalimaud, Champalimaud Foundation, Lisbon, Portugal; 5 Faculté de Médecine Et Médecine Dentaire, Université Catholique de Louvain, Brussels, Belgium; 6 Serviço de Imagiologia, Centro Clínico Champalimaud, Champalimaud Foundation, Lisbon, Portugal; 7 Department of Morphological Sciences, Córdoba University Medical School, Córdoba, Spain; 8 Instituto de Ciências Biomédicas Abel Salazar, Universidade Do Porto, Porto, Portugal; 9 Instituto de Investigação E Inovação Em Saúde, Universidade Do Porto, Porto, Portugal

**Keywords:** Prostate cancer, Retzius-sparing robot-assisted radical prostatectomy, Urinary incontinence, Magnetic resonance imaging, Prognostic tool

## Abstract

The influence of anatomical parameters on urinary continence (UC) after Retzius-sparing robot-assisted radical prostatectomy (RS-RARP) remains uncharted. Our objective was to evaluate their association with UC at 3, 6 and 12 months post-operatively. Data from patients who underwent RS-RARP were prospectively collected. Continence was defined as no pad use. Anatomic variables were measured on preoperative magnetic resonance imaging (MRI). Regression analyses were performed to identify predictors of UC at each time point. We included 158 patients with a median age of 60 years, most of whom had a localized tumor (≤ cT2). On multivariate analyses, at 3 months post-surgery, urinary incontinence (UI) rises with age, odds ratio (OR) 1.07 [95% confidence interval (CI) 1.004−1.142] and with prostate volume (PV), OR 1.029 (95% CI 1.006−1.052); it reduces with longer membranous urethral length (MUL), OR 0.875 (95% CI 0.780−0.983) and with higher membranous urethral volume (MUV), OR 0.299 (95% CI 0.121−0.737). At 6 months, UI rises with PV, OR 1.033 (95% CI 1.011−1.056) and decreases with MUV, OR 0.1504 (95% CI 0.050−0.444). Significantly, at 12 months post-surgery, the only predictor of UI is MUL, OR 0.830 (95% CI 0.706−0.975), establishing a threshold associated with a risk of UI of 5% (MUL > 15 mm) in opposition to a risk of 25% (MUL < 10 mm). This single institutional study requires external validation. To our knowledge, this is the first prospective cohort study supporting MUL as the single independent predictor of UC at 12 months post-surgery. By establishing MUL thresholds, we enable precise patient counseling.

**Supplementary Information:**

The online version contains supplementary material available at 10.1007/s11701-024-01986-8.

## Introduction

Radical prostatectomy (RP) is a treatment option with curative intent in clinically localized prostate cancer (PC) [1]. However, urinary incontinence (UI) represents a significant adverse consequence, detrimentally impacting daily quality of life [2] and fostering surgical regret [3]. Despite a gradual improvement of urinary continence (UC) recovery during the first year after robot-assisted radical prostatectomy (RARP), the rate of UI employing a pad-free definition ranges from 4 to 31%, with a mean value of 16% at 12 months [4]. To preserve UC after RARP, several surgical techniques have been proposed [5]. In contrast to conventional RP, whether performed through open, laparoscopic or robotic techniques, these still reach the prostate anteriorly, dissecting the bladder from the anterior abdominal wall. The novel Retzius-sparing robot-assisted radical prostatectomy (RS-RARP) accesses the prostate from a posterior approach, preserving the integrity of the pubovesical complex and the endopelvic fascia, while maintaining the anterior fixation of the bladder to the abdominal wall [6, 7]. Compared to conventional surgery, RS-RARP is associated with faster UC recovery [8].

The search for predictors of UC recovery through measurable anatomic parameters on preoperative pelvic magnetic resonance imaging (MRI) is work in progress [9, 10]. Among these predictors are prostate volume (PV), membranous urethral length (MUL), membranous urethral volume (MUV), levator ani muscle thickness (LAT), inner (ILD) and outer (OLD) levator ani muscle distance [9–14]. So far, predictors for short-term UC recovery after RS-RARP include PV, pre- and post-operative MUL [15–17]. The impact of measurable anatomic parameters on preoperative MRI for long-term continence outcomes after RS-RARP has yet to be described.

The objectives of the present study include the comprehensive assessment of both clinical and anatomical factors to elucidate their correlation with UC as well as the development of a reliable yet straightforward predictive tool, for estimating the probability of definitive UC recovery. This endeavor requires a longitudinal evaluation of parameters that may impact urinary outcomes.

## Patients and methods

### Patients and functional status evaluation

This study comprises 213 consecutive patients who were evaluated with a preoperative 3.0-Tesla MRI and underwent RS-RARP, both performed at our institution between July 2017 and December 2022. The following parameters were collected prospectively: age, body mass index (BMI), Charlson comorbidity index (CCI), preoperative oncological parameters such as prostate-specific antigen (PSA), highest International Society of Urological Pathology (ISUP) grade group on biopsy, and MRI-based tumor clinical staging using Prostate Imaging-Reporting and Data System Version 2.1 (PI-RADSv2.1) [18]. All patients had localized PC and were evaluated for urinary function preoperatively and postoperatively at 3, 6 and 12 months through pad count, by answering the third question of the Expanded Prostate Cancer Index Composite-26 questionnaire (EPIC-26) [19]. Continence was defined as no pad use. Excluded were those who underwent neoadjuvant or adjuvant radiotherapy (*n* = 6), prior pelvic surgery (*n* = 6) or missing follow-up information for continence (*n* = 43), leaving 158 patients eligible for analysis. Data collection followed the principles outlined in the Declaration of Helsinki. All patients provided written informed consent for study inclusion, approved by the Institutional Ethics Committee (Approval 7.7.2017).

### MRI protocol and measurements

MRI was performed with a 3.0-Tesla system (Ingenia, Philips Healthcare, The Netherlands), equipped with a phased array coil, parallel imaging and multi-transmission radiofrequency capabilities, in the following free-breathing imaging sequences: (a) fast sagittal TSE T2-weighted (TR/TE, 7400 ms/100 ms; FOV, 300 mm × 300 mm; section thickness 5 mm; acquisition time, 36 s) used as localizer; (b) axial high b-value diffusion-weighted (TR/TE, 4000 ms/90 ms; b-values of 0 and 1400 s/mm^2^; FOV, 200 mm × 200 mm; section thickness 4 mm); (c) axial, coronal and sagittal high-resolution TSE T2-weighted (TR/TE, 3000–4000 ms/85–135 ms; FOV, 180–250 mm × 180–300 mm; section thickness 3 mm) and (d) axial TSE T1-weighted (TR/TE, 580 ms/10 ms; FOV, 290 mm × 350 mm; section thickness 5 mm). These were complemented by dynamic contrast-enhanced volumetric gradient echo T1-weighted imaging (TR/TE, 6 ms/3 ms; FOV, 180 mm × 180 mm; section thickness 3 mm).

**Fig. 1:**
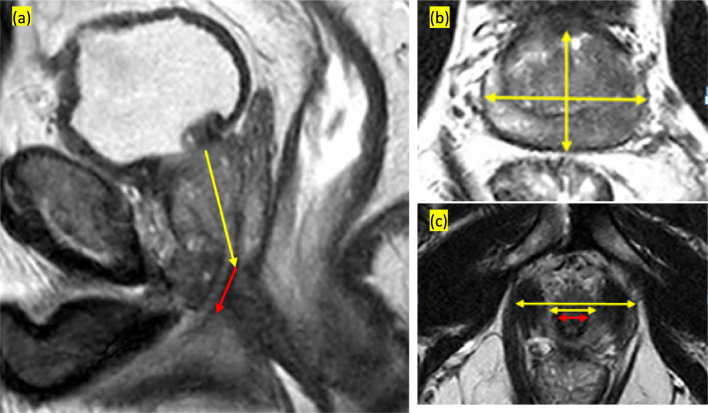
**a** Midsagittal T2-weighted image. The yellow arrow displays maximum height of the prostate, measured from base to apex. The red arrow defines MUL, measured from the apex of the prostate to the upper border of penile bulb, at the dorsal side of the urethral lumen. **b** Axial T2-weighted image at maximal prostate width. The horizontal yellow double arrow shows maximal prostate width and the vertical yellow double arrow displays maximal prostate length. **c** Axial T2-weighted image immediately below the caudal margin of prostate apex. The horizontal red double arrow represents urethral width. The shorter and the longer yellow double arrows display the ILD and OLD, respectively. *ILD* inner levator ani muscle distance, *MUL* membranous urethral length, *OLD* outer levator ani muscle distance

MRI measurements were performed as previously described and standardized [9, 20, 21] in axial and sagittal T2 planes with simultaneous cross-coronal T2 demarcation ensuring measurements were performed in the midsagittal T2 plane as per protocol. These were recorded as follows: (i) PV (ml) = π/6 × maximum height of the prostate × maximal prostate width × maximal prostate length; (ii) maximum height of the prostate measured from base to apex (midsagittal T2-weighted image); (iii) maximal prostate width and maximal prostate length measured at the same axial level, at the level of the maximal prostate width (axial T2-weighted image); (iv) MUL (mm) = distance between the apex of the prostate to the upper border of penile bulb, at the dorsal side of the urethral lumen (sagittal T2-weighted image); (v) urethral width (mm) = maximal diameter of urethra, measured immediately below the caudal margin of prostate apex (axial T2-weighted image); (vi) MUV (ml) calculated as urethral length × π × (urethral width/2)^2^; (vii) ILD (mm) = the narrowest distance from inner border of levator ani muscles immediately below the prostatic apex (axial T2-weighted image); (viii) OLD (mm) = distance from outer border of levator ani muscles measured at same level as ILD (axial T2-weighted image) and (ix) LAT (mm) calculated as (ODL-IDL)/2 (Fig. 1).

MRI measurements were performed by one urologist blinded to all clinical and pathological data. The urologist underwent training under the supervision of an expert radiologist (15 years of experience).

### Operative technique

Surgeries were performed using the 4-arm Da Vinci Xi surgical system (Intuitive Surgical, Sunnyvale, CA). The RS-RARP technique was originally described by Galfano et al. [22]. This posterior surgical approach requires a careful dissection of detrusor fibers from the base of the prostate to prevent ureteral damage, ultimately ensuring the preservation of the bladder neck [23]. Unilateral or bilateral neurovascular excision was undertaken in patients with erectile dysfunction, high risk or locally advanced disease. The anastomosis between the urethra and the bladder was performed with a running, barbed 3/0 polyglyconate suture (V-LOC^®^; Covidien, Mansfield, MA). The integrity of the urethrovesical anastomosis was confirmed intraoperatively with intravesical instillation of 150 ml of sterile saline through a 16F Foley catheter.

### Pathology examination

The same senior pathologist reviewed prostatic biopsies and surgical specimens. The ISUP consensus and the American Joint Committee on Cancer 8th edition schemes were followed for grading and staging.

### Statistical analysis

Quantitative variables were reported as medians and interquartile range (IQR), and qualitative variables as counts or percentages. We employed univariate logistic regression analysis to evaluate possible significant associations between patients’ demographic and clinical factors, preoperative MRI parameters, intraoperative surgical variables, and UI at 3, 6 and 12 months post-surgery. We then used multivariable logistic regression analysis to assess independent significant predictors of continence at those time points. Finally, we established a comprehensive model for estimating the probability of UC recovery which only included variables that exhibited independent statistical significance in the multivariate analysis, based on a “Backward Conditional” method. The quality of the models at each time point was evaluated based on the area under the receiver operating characteristic curve (AUC).

All statistical analyses were performed using IBM^®^ SPSS^®^ Statistics version 27 for Windows, the level of significance was set at *p* < 0.05 and a CI of 95% was assumed.

## Results

The median patient age was 60 years, median BMI 27 kg/m^2^ and 86% of the patients had a CCI ≤ 2. Of note, median PV was 32.7 ml (IQR 25.1−43.3), median MUL 15.1 mm (IQR 12.1−17.4) and median MUV 1.2 ml (IQR 0.9−1.6). Remaining patient and tumor characteristics as well as MRI measurements are presented in Table 1.

**Table 1:** Patient and tumor characteristics, and MRI anatomic parameters

Patient characteristics	Full cohort
Patients evaluated, *n*	158
Age (years), median (IQR)	60 (58–68)
BMI (kg/m^2^), median (IQR)	27 (25–29)
CCI, *n* (%)	
≤ 1	52 (33)
2	84 (53)
≥ 3	22 (14)
Preoperative PSA (ng/ml) median (IQR)	6 (5–8)
Highest biopsy ISUP grade group, *n* (%)	
GG 1	13 (8)
GG 2	95 (60)
GG 3	44 (28)
GG 4	5 (3)
GG 5	1 (1)
MRI-based T stage, *n* (%)	
cT1	19 (12)
cT2	107 (68)
cT3a	27 (17)
cT3b	5 (3)
Preoperative MRI variables, median (IQR)	
PV (ml)	32.7 (25.1–43.3)
MUL (mm)	15.1 (12.1–17.4)
Urethral width (mm)	11.2 (10.0–12.6)
MUV (ml)	1.2 (0.9–1.6)
LAT (mm)	10.1 (8.6–11.1)
ILD (mm)	16.7 (15.1–19.1)
OLD (mm)	37.2 (34.0–39.2)
Patients with recorded daily pad use, *n* (%)	
No pad use	151 (100)
Pad use	0 (0)

*BMI* body mass index, *CCI* Charlson comorbidity index, *PSA* prostate-specific antigen, *ISUP* International Society of Urological Pathology, *GG* grade group, *MRI* magnetic resonance imaging, *IQR* interquartile range, *PV* prostate volume, *MUL* membranous urethral length, *MUV* membranous urethral volume, *LAT* levator ani muscle thickness, *ILD* inner levator ani muscle distance, *OLD* outer levator ani muscle distance

Pathological assessment revealed locally advanced disease in 31% of the patients, and the rate of positive surgical margins (over 3 mm in length) was 12%. A total of 59% of the patients underwent bilateral nerve-sparing, while bladder neck preservation was performed in 97%. Additional surgical variables and tumor pathological staging are presented in Table 2.

**Table 2:** Surgical variables and tumor pathological staging

Patient characteristics	Full cohort
Patients evaluated, *n*	158
Patients with recorded nerve-sparing status, *n*	150
No nerve-sparing, *n (%)*	21 (14)
Unilateral, *n (%)*	41 (27)
Bilateral, *n (%)*	88 (59)
Patients with recorded bladder neck dissection status, *n*	116
Preserved, *n (%)*	113 (97)
Not preserved,* n (%)*	3 (3)
Lymph node dissection	
Lymphadenectomy,* n (%)*	50 (32)
No lymphadenectomy, *n (%)*	108 (68)
Lymph nodes removed, median (IQR)	23 (19–30)
Postoperative complications (Clavien–Dindo > II)	1 (0.6)
Lymphocele drainage, *n* (%)	1 (0.6)
ISUP on specimen (grade group)	
GG 1, *n* (%)	6 (4)
GG 2, *n* (%)	113 (72)
GG 3, *n* (%)	37 (23)
GG 4, *n* (%)	2 (1)
GG 5, *n* (%)	0
Pathological T stage (pT)	
pT2, *n* (%)	109 (69)
pT3a, *n* (%)	39 (25)
pT3b, *n* (%)	10 (6)
Surgical margin	
Overall positive, *n* (%)	48 (30)
Positive, with > 3 mm total length extension, *n* (%)	19 (12)
N stage	
pNx, *n* (%)	108 (68)
pN0, *n* (%)	43 (27)
pN1, *n* (%)	6 (4)
PSA persistence and recurrence	
PSA ≥ 0.2 ng/ml at 3 months, *n* (%)	8 (5)
PSA ≥ 0.2 ng/ml at 12 months, *n* (%)	12 (8)

*IQR* interquartile range, *ISUP* International Society of Urological Pathology, *GG* grade group, *PSA* prostate-specific antigen

**Fig. 2:**
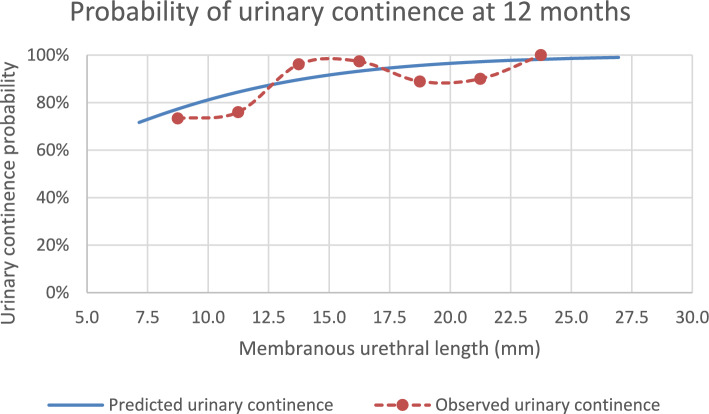
The blue line represents the probability of a patient being urinary continent at 12 months after Retzius-sparing robot-assisted radical prostatectomy, based on the logistic regression model. The orange dots represent the observed proportion of urinary continent patients, calculated according to 2.5 mm MUL intervals

Concerning UC, it improved with time, 63.3%, 77.9% and 89.1% of the patients were pad-free at 3, 6 and 12 months post-surgery, respectively. Univariate analysis demonstrated that age, PV, MUL, MUV and CCI were significant predictors for UI at 3 months; PV, MUL and MUV were significant predictors for UI at 6 months; MUL was the only significant predictor of UI at 12 months (Table 3). Multivariate analysis revealed that except for CCI, the same variables remained significant at 3 months. However, by 6 months, age and MUL had lost significance (Table 4). Regarding the performance of the multivariate models at 3, 6 and 12 months, the AUC was 0.782 (CI 0.707–0.856), 0.783 (CI 0.700–0.865) and 0.690 (CI 0.529−0.850), respectively. Our prediction model for UC recovery at 3 months after RS-RARP includes age, PV, MUL and MUV. At 6 months, the model includes PV and MUV, while at 12 months, MUL stands as the sole predictor. The prediction model and tool are available in the supplementary material. At 12 months, the risk of UI with MUL < 10 mm was 25%. Conversely, the risk of UI with MUL > 15 mm was 5% (Fig. 2).

**Table 3:** Univariate logistic regression of factors predicting post-operative urinary incontinence at 3, 6 and 12 months

	3 months		6 months		12 months	
	OR (95% CI)	*p* value	OR (95% CI)	*p* value	OR (95% CI)	*p* value
Age (years)	1.090 (1.029–1.154)	0.003	1.062 (0.997–1.133)	0.064	1.051 (0.960–1.150)	0.283
BMI (kg/m^2^)	1.208 (0.761–1.919)	0.422	1.004 (0.598–1.712)	0.987	0.748 (0.342–1.635)	0.467
CCI		0.047		0.203		0.265
2*	1.941 (0.903–4.173)	0.089	1.303 (0.536–3.165)	0.559	3.563 (0.755–1.681)	0.108
≥ 3*	3.600 (1.262–10.267)	0.017	2.730 (0.884–8.429)	0.081	2.300 (0.302–17.496)	0.421
PSA (ng/ml)	1.034 (0.949–1.128)	0.445	1.057 (0.962–1.162)	0.250	1.041 (0.913–1.187)	0.549
MRI-based T stage		0.936		0.621		0.923
cT2**	1.346 (0.474–3.819)	0.577	2.728 (0.591–12.604)	0.199	No sense values	0.998
cT3a**	1.490 (0.433–5.121)	0.527	2.975 (0.544–16.273)	0.209	No sense values	0.998
cT3b**	No sense values	0.999	No sense values	0.999	No sense values	1.000
Preoperative MRI variables						
PV (ml)	1.027 (1.008–1.046)	0.005	1.026 (1.007–1.045)	0.008	1.005 (0.979–1.024)	0.695
MUL (mm)	0.832 (0.754–0.918)	< 0.001	0.861 (0.772–0.961)	0.007	0.830 (0.706–0.975)	0.024
Urethral width (mm)	0.889 (0.750–1.053)	0.173	0.857 (0.700–1.049)	0.135	0.976 (0.741–1.284)	0.862
MUV (ml)	0.234 (0.106–0.518)	< 0.001	0.182 (0.066–0.504)	0.001	0.345 (0.097–1.235)	0.102
LAT (mm)	1.086 (0.912–1.294)	0.355	1.070 (0.874–1.311)	0.511	1.037 (0.777–1.384)	0.806
ILD (mm)	1.009 (0.911–1.118)	0.865	1.024 (0.911–1.152)	0.689	1.062 (0.906–1.246)	0.455
Nerve sparing		0.641		0.976		0.434
Unilateral***	1.191 (0.454–3.126)	0.722	1.135 (0.368–3.497)	0.826	0.611 (0.071–5.248)	0.655
No nerve sparing***	0.737 (0.336–1.617)	0.447	1.021 (0.416–2.505)	0.963	1.886 (0.590–6.023)	0.284

*with reference to CCI ≤ 1; **with reference to cT1; ***with reference to bilateral nerve-sparing

*BMI* body mass index, *CCI* Charlson comorbidity index, *CI* confidence interval, *GG* grade group, *ILD* inner levator ani muscle distance, *LAT* levator ani muscle thickness, *MRI* magnetic resonance imaging, *MUL* membranous urethral length, *MUV* membranous urethral volume, *OR* odds ratio, *PSA* prostate-specific antigen, *PV* prostate volume

**Table 4:** Multivariate logistic regression of factors predicting post-operative urinary incontinence at 3, 6 and 12 months

	3 months		6 months		12 months	
	OR (95% CI)	*p* value	OR (95% CI)	*p* value	OR (95% CI)	*p* value
Age (years)	1.071 (1.004–1.142)	0.037				
PV (ml)	1.029 (1.006–1.052)	0.012	1.033 (1.011–1.056)	0.003		
MUL (mm)	0.875 (0.780–0.983)	0.024			0.830 (0.706–0.975)	0.024
MUV (ml)	0.299 (0.121–0.737)	0.009	0.150 (0.05–0.444)	< 0.001		

*CI* confidence interval, *MUL* membranous urethral length, *MUV* membranous urethral volume, *OR* odds ratio, *PV* prostate volume

## Discussion

The advantageous impact of RS-RARP over conventional RARP on early UC recovery has been established in a landmark study in which at 3 and 12 months post-surgery, 75.7% (95% CI 64.6%−86.9%) and 95.8% (95% CI 90.4%−100%) of patients were pad free, accordingly [8]. Early on, Chang et al. established through post-operative cystourethrography, that RS-RARP was associated with less bladder neck descent and improved early continence outcome [24]. Several randomized controlled trials have established similar outcomes [25–27], alike our dataset, where the rate of pad-free patients was 63.3% and 89.1% at 3 and 12 months post-surgery, respectively. The rate of UC recovery consistently slows after the first three months. Consequently, the development of a predictive tool for UC recovery within the 3-month timeframe holds significance, as well as the definitive long-term status by the 12-month mark [28].

Predictive models are also useful for postoperative treatment planning. Conservative treatment, including pelvic floor stimulation and biofeedback, can be offered earlier for patients at high risk of developing long-lasting UI. Such a proactive strategy may help patients who have the potential for recovery [29]. We developed a straightforward prediction tool for continence after RS-RARP, incorporating clinical factors and anatomic parameters. We omitted predictors related to surgeon’s experience based on our prior research, which highlighted the robustness of RS-RARP for UC recovery regardless of surgeon experience [30]. In our data set, age, PV, MUL and MUV are significant predictors for continence at 3 months. The AUC of the model stands at 78.2%, indicating a moderate level of accuracy. Several studies and meta-analyses [10, 31, 32] have also demonstrated that MUL is the most studied and reliable urethral parameter to integrate a prediction tool [21]. Our investigation similarly demonstrated the significant contribution of a longer MUL to UC recovery. Most importantly, we have been able to find a threshold that enables pre-operative patient counselling regarding definitive UC recovery.

When considering several surgical approaches (open, laparoscopic and robotic prostatectomy), multivariate analysis confirmed that age and MUL were significant independent predictors for early UC recovery in a patient population with a median age of 71 years old [33]. Wang et al. also established that patients with a longer MUL had better UC recovery at one and 3 months post-RARP [34]. In another study, at 12 months post-surgery, unlike our findings, age has been described to impact continence upon univariate analysis [11]. Nonetheless, the latter study involved patients subjected to conventional RARP, once again potentially accounting for the observed discrepancy. However, in alignment with our data, both MUL and surgical experience, rather than patient age, have been described as predictors of UI at 12 months following RARP [35]. Surgical experience is a recognized predictive factor for UI following RARP [4], yet its predictive ability does not extend to RS-RARP [30]. Even though PV has been correlated with age and a protruded median lobe (*p* < 0.001) [36], the latter has also been described to impact early postoperative UC recovery [37]. In our study, PV is a significant predictor of early UC recovery independently of age, and we highlight its usefulness as already integrated in the PI-RADSv2.1 protocol [18]. Lastly, our lack of association between BMI and comorbidity is not unexpected given that these are also absent following RP [38].

Within our dataset, the parameters of LAT, ILD, and OLD measured in the axial T2-weighted sequence did not emerge as statistically significant predictors of UC recovery at any time point. Different measurement standards and surgical techniques may assign varying degrees of importance to identical predictors for UC recovery [39]. For example, LAT measured on an axial T2-weighted sequence was described as an independent predictor of immediate UC recovery following laparoscopic radical prostatectomy [40]. When measured on a coronal T2-weighted sequence it was found to also behave as an independent predictor of UC recovery three months after RARP [12]. However, in line with our study, a recent systematic review failed to identify any connection between LAT and long-term UC recovery [41].

Bladder neck and neurovascular bundle (NVB) preservation were not significantly associated with post-operative UC and indeed they have not consistently been reported as predictors of UC recovery [38, 42, 43]. During RS-RARP the detrusor is carefully peeled away on the posterior bladder neck to avoid ureteral lesions and the bladder neck is almost always preserved [36]. In our series, it was preserved in 97% of patients, preventing conclusions as regards the impact of bladder resection on postoperative UI.

We acknowledge that MRI measurement might have moderate interrater agreement which is a bias for the generalization of our results based on a single rater [39, 44], an issue which may soon be solved by automated MRI measurements [45]. We attempted to mitigate this limitation by following a standardized protocol for midsagittal MUL measurements [20, 21] and as such we believe it does not affect our conclusions [39]. Other limitations include a pre-operative lack of assessment for the presence of lower urinary tract symptoms which could affect UC recovery, a single institution perspective and a limited number of patients. Finally, our findings require additional external validation.

## Conclusions

To the best of our knowledge, we have developed the first tool to predict individual postoperative UI rates following RS-RARP, using preoperative clinical and measurable anatomical features on preoperative MRI. Besides providing valuable guidance for counseling, it also draws the attention of the surgeon toward anatomic parameters which are not routinely reported on the PI-RADSv2.1 protocol [18] but are included in the prediction model. Our prediction tool is user-friendly, has acceptable accuracy, and is intended to help urologists estimate the individual probability of UC recovery, defined as pad-free status, at 3 months post-surgery. Furthermore, we highlight the importance of MUL as the single independent predictor of continence at 12 months. By establishing MUL thresholds for UC recovery, we enable precise patient counseling, provide realistic expectations and allow informed choices that enhance quality of life.

## Supplementary Information

Below is the link to the electronic supplementary material.

Supplementary file1 (DOCX 62 KB)

Supplementary file2 (XLSX 23 KB)

## Data Availability

Data will be made available upon reasonable request.

## References

[1] Mottet N, van den Bergh RCN, Briers E, Van den Broeck T, Cumberbatch MG, De Santis M et al (2021) EAU-EANM-ESTRO-ESUR-SIOG guidelines on prostate cancer—2020 update. Part 1: screening, diagnosis, and local treatment with curative intent. Eur Urol 79:243–262. 10.1016/j.eururo.2020.09.042 33172724 10.1016/j.eururo.2020.09.042

[2] Lehto US, Tenhola H, Taari K, Aromaa A (2017) Patients’ perceptions of the negative effects following different prostate cancer treatments and the impact on psychological well-being: a nationwide survey. Br J Cancer 116:864–873. 10.1038/bjc.2017.30 28222069 10.1038/bjc.2017.30 PMC5379142

[3] Fanshawe JB, Wai-Shun Chan V, Asif A, Ng A, Van Hemelrijck M, Cathcart P et al (2023) Decision regret in patients with localised prostate cancer: a systematic review and meta-analysis. Eur Urol Oncol. 10.1016/j.euo.2023.02.005 36870852 10.1016/j.euo.2023.02.005

[4] Ficarra V, Novara G, Rosen RC, Artibani W, Carroll PR, Costello A et al (2012) Systematic review and meta-analysis of studies reporting urinary continence recovery after robot-assisted radical prostatectomy. Eur Urol 62:405–417. 10.1016/j.eururo.2012.05.045 22749852 10.1016/j.eururo.2012.05.045

[5] Arroyo C, Martini A, Wang J, Tewari AK (2019) Anatomical, surgical and technical factors influencing continence after radical prostatectomy. Ther Adv Urol 11:175628721881378. 10.1177/1756287218813787 10.1177/1756287218813787 PMC6329031 30671134

[6] Kadono Y, Nohara T, Kawaguchi S, Iwamoto H, Yaegashi H, Shigehara K et al (2022) Impact of pelvic anatomical changes caused by radical prostatectomy. Cancers (Basel). 10.3390/cancers14133050 35804823 10.3390/cancers14133050 PMC9265134

[7] Asimakopoulos AD, Miano R, Galfano A, Bocciardi AM, Vespasiani G, Spera E et al (2015) Retzius-sparing robot-assisted laparoscopic radical prostatectomy: critical appraisal of the anatomic landmarks for a complete intrafascial approach. Clin Anat 28:896–902. 10.1002/ca.22576 26194970 10.1002/ca.22576

[8] Menon M, Dalela D, Jamil M, Diaz M, Tallman C, Abdollah F et al (2018) Functional recovery, oncologic outcomes and postoperative complications after robot-assisted radical prostatectomy: an evidence-based analysis comparing the Retzius sparing and standard approaches. J Urol 199:1210–1217. 10.1016/j.juro.2017.11.115 29225060 10.1016/j.juro.2017.11.115

[9] Von Bodman C, Matsushita K, Savage C, Matikainen MP, Eastham JA, Scardino PT et al (2012) Recovery of urinary function after radical prostatectomy: predictors of urinary function on preoperative prostate magnetic resonance imaging. J Urol 187:945–950. 10.1016/j.juro.2011.10.143 22264458 10.1016/j.juro.2011.10.143 PMC4768862

[10] van Dijk-de Haan MC, Boellaard TN, Tissier R, Heijmink SWTPJ, van Leeuwen PJ, van der Poel HG et al (2022) Value of different magnetic resonance imaging-based measurements of anatomical structures on preoperative prostate imaging in predicting urinary continence after radical prostatectomy in men with prostate cancer: a systematic review and meta-analysis. Eur Urol Focus 8:1211–1225. 10.1016/j.euf.2022.01.015 35181284 10.1016/j.euf.2022.01.015

[11] Kim LHC, Patel A, Kinsella N, Sharabiani MTA, Ap Dafydd D, Cahill D (2019) Association between preoperative magnetic resonance imaging–based urethral parameters and continence recovery following robot-assisted radical prostatectomy. Eur Urol Focus 6:1–8. 10.1016/j.euf.2019.01.011 30691961 10.1016/j.euf.2019.01.011

[12] Tutolo M, Rosiello G, Stabile G, Tasso G, Oreggia D, De Wever L et al (2022) The key role of levator ani thickness for early urinary continence recovery in patients undergoing robot-assisted radical prostatectomy: a multi-institutional study. Neurourol Urodyn. 10.1002/nau.25001 35781824 10.1002/nau.25001

[13] Grivas N, van der Roest R, Schouten D, Cavicchioli F, Tillier C, Bex A et al (2018) Quantitative assessment of fascia preservation improves the prediction of membranous urethral length and inner levator distance on continence outcome after robot-assisted radical prostatectomy. Neurourol Urodyn 37:417–425. 10.1002/nau.23318 28586158 10.1002/nau.23318

[14] Heesakkers J, Farag F, Bauer RM, Sandhu J, De Ridder D, Stenzl A (2017) Pathophysiology and contributing factors in postprostatectomy incontinence: a review. Eur Urol 71:936–944. 10.1016/j.eururo.2016.09.031 27720536 10.1016/j.eururo.2016.09.031

[15] Galfano A, Panarello D, Secco S, Di Trapani D, Barbieri M, Napoli G et al (2018) Does prostate volume have an impact on the functional and oncological results of Retzius-sparing robot-assisted radical prostatectomy. Minerva Urol e Nefrol. 10.23736/S0393-2249.18.03069-2 10.23736/S0393-2249.18.03069-2 29595042

[16] Li Y, Li W, Lu W, Chen M, Gao J, Yang Y et al (2020) Association of preoperative urethral parameters on magnetic resonance imaging and immediate recovery of continence following Retzius-sparing robot-assisted radical prostatectomy. Transl Androl Urol 9:501–509. 10.21037/tau.2019.12.17 32420156 10.21037/tau.2019.12.17 PMC7215013

[17] Ota Y, Hamamoto S, Matsuyama N, Hamakawa T, Iwatsuki S, Etani T et al (2021) Pelvic anatomical features after Retzius-sparing robot-assisted radical prostatectomy intended for early recovery of urinary symptoms. J Endourol 35:296–304. 10.1089/end.2020.0463 32935558 10.1089/end.2020.0463

[18] Turkbey B, Rosenkrantz AB, Haider MA, Padhani AR, Villeirs G, Macura KJ et al (2019) Prostate imaging reporting and data system version 2.1: 2019 update of prostate imaging reporting and data system version 2. Eur Urol 2019(76):340–351. 10.1016/j.eururo.2019.02.033 10.1016/j.eururo.2019.02.033 30898406

[19] Szymanski KM, Wei JT, Dunn RL, Sanda MG (2010) Development and validation of an abbreviated version of the expanded prostate cancer index composite instrument for measuring health-related quality of life among prostate cancer survivors. Urology 76:1245–1250. 10.1016/j.urology.2010.01.027 20350762 10.1016/j.urology.2010.01.027 PMC3152317

[20] Veerman H, Hagens MJ, Hoeks CM, van der Poel HG, van Leeuwen PJ, Vis AN et al (2022) A standardized method to measure the membranous urethral length (MUL) on MRI of the prostate with high inter- and intra-observer agreement. Eur Radiol. 10.1007/s00330-022-09320-2 36512044 10.1007/s00330-022-09320-2

[21] Boellaard TN, van Dijk-de Haan MC, Heijmink SWTPJ, Tillier CN, Veerman H, Mertens LS et al (2023) Membranous urethral length measurement on preoperative MRI to predict incontinence after radical prostatectomy: a literature review towards a proposal for measurement standardization. Eur Radiol. 10.1007/s00330-023-10180-7 37737870 10.1007/s00330-023-10180-7 PMC10957670

[22] Galfano A, Ascione A, Grimaldi S, Petralia G, Strada E, Bocciardi AM (2010) A new anatomic approach for robot-assisted laparoscopic prostatectomy: a feasibility study for completely intrafascial surgery. Eur Urol 58:457–461. 10.1016/j.eururo.2010.06.008 20566236 10.1016/j.eururo.2010.06.008

[23] Nyarangi-Dix JN, Tichy D, Hatiboglu G, Pahernik S, Tosev G, Hohenfellner M (2018) Complete bladder neck preservation promotes long-term post-prostatectomy continence without compromising midterm oncological outcome: analysis of a randomised controlled cohort. World J Urol 36:349–355. 10.1007/s00345-017-2134-1 29214353 10.1007/s00345-017-2134-1

[24] Chang L-W, Hung S-C, Hu J-C, Chiu K-Y (2018) Retzius-sparing robotic-assisted radical prostatectomy associated with less bladder neck descent and better early continence outcome. Anticancer Res 38:345–351 29277793 10.21873/anticanres.12228

[25] Dalela D, Jeong W, Prasad M-A, Sood A, Abdollah F, Diaz M et al (2017) A pragmatic randomized controlled trial examining the impact of the Retzius-sparing approach on early urinary continence recovery after robot-assisted radical prostatectomy. Eur Urol 72:677–685. 10.1016/j.eururo.2017.04.029 28483330 10.1016/j.eururo.2017.04.029

[26] Asimakopoulos AD, Topazio L, De Angelis M, Agrò EF, Pastore AL, Fuschi A et al (2018) Retzius-sparing versus standard robot-assisted radical prostatectomy: a prospective randomized comparison on immediate continence rates. Surg Endosc. 10.1007/s00464-018-6499-z 30426256 10.1007/s00464-018-6499-z

[27] Qiu X, Li Y, Chen M, Xu L, Guo S, Marra G et al (2020) Retzius-sparing robot-assisted radical prostatectomy improves early recovery of urinary continence: a randomized, controlled, single-blind trial with a 1-year follow-up. BJU Int. 10.1111/bju.15195 32741099 10.1111/bju.15195

[28] Li X, Zhang H, Jia Z, Wang Y, Song Y, Liao L et al (2020) Urinary continence outcomes of four years of follow-up and predictors of early and late urinary continence in patients undergoing robot-assisted radical prostatectomy. BMC Urol 20:1–10. 10.1186/s12894-020-00601-w 32188426 10.1186/s12894-020-00601-w PMC7079466

[29] Castellan P, Ferretti S, Litterio G, Marchioni M, Schips L (2023) Management of urinary incontinence following radical prostatectomy: challenges and solutions. Ther Clin Risk Manag 19:43–56. 10.2147/TCRM.S283305 36686217 10.2147/TCRM.S283305 PMC9851058

[30] Fonseca J, Froes G, Moraes-Fontes MF, Rebola J, Lúcio R, Almeida M et al (2023) Urinary continence recovery after Retzius-sparing robot-assisted radical prostatectomy in relation to surgeon experience. J Robot Surg. 10.1007/s11701-023-01687-8 37528286 10.1007/s11701-023-01687-8 PMC10492722

[31] Mungovan SF, Sandhu JS, Akin O, Smart NA, Graham PL, Patel MI (2017) Preoperative membranous urethral length measurement and continence recovery following radical prostatectomy: a systematic review and meta-analysis. Eur Urol 71:368–378. 10.1016/j.eururo.2016.06.023 27394644 10.1016/j.eururo.2016.06.023 PMC5600894

[32] Mac Curtain BM, Sugrue DD, Qian W, O’Callaghan M, Davis NF (2023) Membranous urethral length and urinary incontinence following robot-assisted radical prostatectomy: a systematic review and meta-analysis. BJU Int. 10.1111/bju.16170 37667431 10.1111/bju.16170

[33] Park S, Byun J (2021) A study of predictive models for early outcomes of post-prostatectomy incontinence: machine learning approach vs. Logistic regression analysis approach. Appl Sci. 10.3390/app11136225

[34] Wang M, Deng R, Wang L, Li M, Zeng T, Na Y et al (2024) Association between 3D membranous urethral parameters and urinary continence recovery after RARP. Eur J Med Res 29:1–8. 10.1186/s40001-024-01758-y 38475943 10.1186/s40001-024-01758-y PMC10929111

[35] Yamashita K, Kijima Y, Sekido E, Nagasaka N, Inui M (2023) Predictors of long-term urinary incontinence after robot-assisted laparoscopic prostatectomy. Res Reports Urol 15:387–393. 10.2147/RRU.S419903 10.2147/RRU.S419903 PMC10455970 37638328

[36] Qian J, Fu Y, Wu X, Xu L, Zhang M, Zhang Q et al (2021) Impact of protruded median lobe on perioperative, urinary continence and oncological outcomes of Retzius-sparing robot-assisted radical prostatectomy. Transl Androl Urol 10:538–547 33718056 10.21037/tau-20-1229 PMC7947452

[37] Hikita K, Honda M, Teraoka S, Nishikawa R, Kimura Y, Tsounapi P et al (2020) Intravesical prostatic protrusion may affect early postoperative continence undergoing robot-assisted radical prostatectomy. BMC Urol 20:4–11. 10.1186/s12894-020-00740-0 33087082 10.1186/s12894-020-00740-0 PMC7579942

[38] Lardas M, Grivas N, Debray TPA, Zattoni F, Berridge C, Cumberbatch M et al (2022) Patient- and tumour-related prognostic factors for urinary incontinence after radical prostatectomy for nonmetastatic prostate cancer: a systematic review and meta-analysis. Eur Urol Focus 8:674–689. 10.1016/j.euf.2021.04.020 33967010 10.1016/j.euf.2021.04.020

[39] Muñoz-Calahorro C, Parada-Blázquez MJ, García-Sánchez C, López-Arellano L, Vizcaíno-Velázquez P, Medina-López RA (2023) Inter-observer variability in male pelvic-floor MRI measurements that might predict post-prostatectomy incontinence. World J Urol 41:1147–1155. 10.1007/s00345-023-04320-3 36795146 10.1007/s00345-023-04320-3

[40] Gu Z, Zheng Z, Zhang W, Mao S, Wang S, Geng J et al (2023) The development and assessment of a predicting nomogram for the recovery of immediate urinary continence following laparoscopic radical prostatectomy. Front Surg 9:1–14. 10.3389/fsurg.2022.1071093 10.3389/fsurg.2022.1071093 PMC9852533 36684134

[41] Muñoz-Calahorro C, García-Sánchez C, Barrero-Candau R, García-Ramos JB, Rodríguez-Pérez AJ, Medina-López RA (2021) Anatomical predictors of long-term urinary incontinence after robot-assisted laparoscopic prostatectomy: a systematic review. Neurourol Urodyn 40:1089–1097. 10.1002/nau.24652 33851426 10.1002/nau.24652

[42] Reeves F, Preece P, Kapoor J, Everaerts W, Murphy DG, Corcoran NM et al (2015) Preservation of the neurovascular bundles is associated with improved time to continence after radical prostatectomy but not long-term continence rates: results of a systematic review and meta-analysis. Eur Urol 68:692–704. 10.1016/j.eururo.2014.10.020 25454614 10.1016/j.eururo.2014.10.020

[43] Liao PC, Hung SC, Hu JC, Chiu KY (2020) Retzius-sparing robotic-assisted radical prostatectomy facilitates early continence regardless of neurovascular bundle sparing. Anticancer Res 40:4075–4080. 10.21873/anticanres.14405 32620655 10.21873/anticanres.14405

[44] Lamberg H, Shankar PR, Singh K, Caoili EM, George AK, Hackett C et al (2022) Preoperative prostate MRI predictors of urinary continence following radical prostatectomy. Radiology. 10.1148/radiol.210500 35040671 10.1148/radiol.210500 PMC8962824

[45] Boellaard TN, Hagens MJ, Veerman H, Yakar D, Mertens LS, Heijmink SWTPJ et al (2023) Prostate MRI for improving personalized risk prediction of incontinence and surgical planning: the role of membranous urethral length measurements and the use of 3D models. Life. 10.1148/radiol.210500 36983985 10.3390/life13030830 PMC10054694

