# Supplementary material for: Membranous urethral length is the single independent predictor of urinary continence recovery at 12 months following Retzius-sparing robot-assisted radical prostatectomy
Source: J Robot Surg. 2024 May 29;18(1):230. doi: 10.1007/s11701-024-01986-8 (PMC11136784; doi:10.1007/s11701-024-01986-8)
Supplement: Supplementary file 1 — Supplementary file1 (DOCX 62 KB) [file 11701_2024_1986_MOESM1_ESM.docx]

**Table S1:** Urinary incontinence at 3 months logistic regression model.

|  | | B | S.E. | Wald | df | Sig. | Exp(B) | 95% C.I. for EXP(B) | |
| --- | --- | --- | --- | --- | --- | --- | --- | --- | --- |
|  |  |  |  |  |  |  |  | Lower | Upper |
| **Variables in the equation** | Age | 0.068 | 0.033 | 4.350 | 1 | 0.037 | 1.071 | 1.004 | 1.142 |
|  | PV | 0.028 | 0.011 | 6.372 | 1 | 0.012 | 1.029 | 1.006 | 1.052 |
|  | MUL | -0.133 | 0.059 | 5.094 | 1 | 0.024 | 0.875 | 0.780 | 0.983 |
|  | MUV | -1.209 | 0.461 | 6.885 | 1 | 0.009 | 0.299 | 0.121 | 0.737 |
|  | Constant | -2.467 | 2.129 | 1.343 | 1 | 0.247 | 0.085 |  |  |


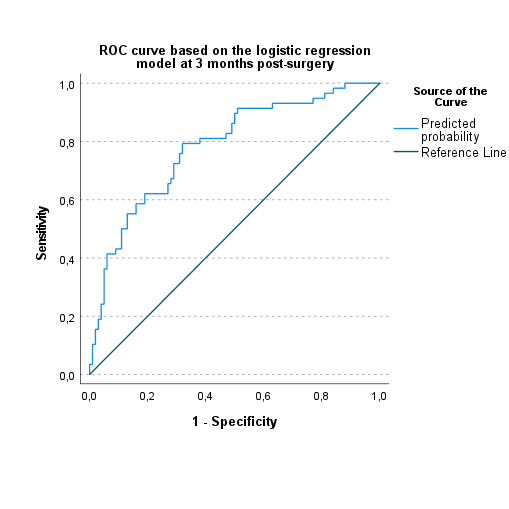


| **Area under the ROC curve (3 months post-surgery)** | | | | |
| --- | --- | --- | --- | --- |
| Area | Std. Error | Asymptotic Sig. | Asymptotic 95% Confidence Interval | |
|  |  |  | Lower Bound | Upper Bound |
| 0.782 | 0.038 | <0.001 | 0.707 | 0.856 |

**Figure S1:** ROC analysis of the logistic regression model developed to predict urinary incontinence at 3 months post-surgery.

**Table S2:** Urinary incontinence at 6 months logistic regression model.

|  | | B | S.E. | Wald | df | Sig. | Exp(B) | 95% C.I. for EXP(B) | |
| --- | --- | --- | --- | --- | --- | --- | --- | --- | --- |
|  |  |  |  |  |  |  |  | Lower | Upper |
| **Variables in the equation** | PV | 0.033 | 0.011 | 8.773 | 1 | 0.003 | 1.033 | 1.011 | 1.056 |
|  | MUV | -1.899 | 0.555 | 11.709 | 1 | 0.001 | 0.150 | 0.050 | 0.444 |
|  | Constant | -0.302 | 0.692 | 0.191 | 1 | 0.662 | 0.739 |  |  |


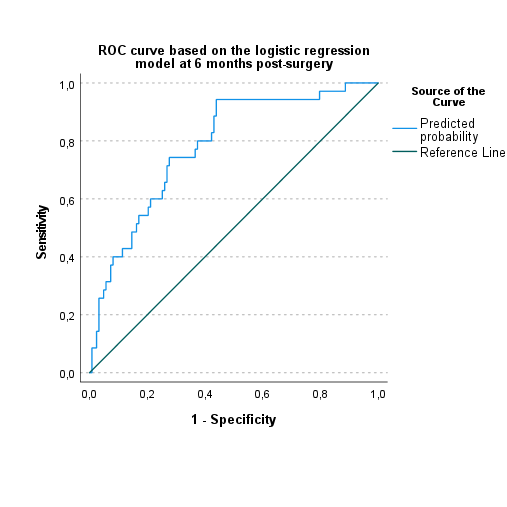


| **Area under the ROC curve (6 months post-surgery)** | | | | |
| --- | --- | --- | --- | --- |
| Area | Std. Error | Asymptotic Sig. | Asymptotic 95% Confidence Interval | |
|  |  |  | Lower Bound | Upper Bound |
| 0.783 | 0.042 | <0.001 | 0.700 | 0.865 |

**Figure S2:** ROC analysis of the logistic regression model developed to predict urinary incontinence at 6 months post-surgery.

**Table S3:** Urinary incontinence at 12 months logistic regression model.

|  | | B | S.E. | Wald | df | Sig. | Exp(B) | 95% C.I. for EXP(B) | |
| --- | --- | --- | --- | --- | --- | --- | --- | --- | --- |
|  |  |  |  |  |  |  |  | Lower | Upper |
| **Variables in the equation** | MUL | -0.187 | 0.082 | 5.130 | 1 | 0.024 | 0.830 | 0.706 | 0.975 |
|  | Constant | 0.408 | 1.114 | 0.134 | 1 | 0.714 | 1.504 |  |  |


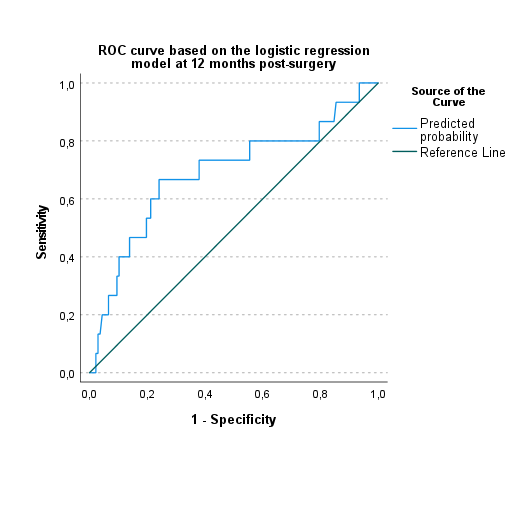


| **Area under the ROC curve (12 months post-surgery)** | | | | |
| --- | --- | --- | --- | --- |
| Area | Std. Error | Asymptotic Sig. | Asymptotic 95% Confidence Interval | |
|  |  |  | Lower Bound | Upper Bound |
| 0.690 | 0.082 | 0.021 | 0.529 | 0.850 |

**Figure S3:** ROC analysis of the logistic regression model developed to predict urinary incontinence at 12 months post-surgery.
